# Trends in gabapentinoid prescribing: A nationwide Danish drug utilization study

**DOI:** 10.1002/bcp.70060

**Published:** 2025-04-09

**Authors:** Anton Pottegård, Lotte Rasmussen, Morten Olesen, Anne Mette Skov Sørensen, Zandra Nymand Ennis, Joseph Kane, Sarah Baxter, Blánaid Hicks

**Affiliations:** ^1^ Clinical Pharmacology and Pharmacy, Department of Public Health University of Southern Denmark Odense Denmark; ^2^ Department of Clinical Pharmacology Copenhagen University Hospital Bispebjerg and Frederiksberg Copenhagen Denmark; ^3^ Department of Clinical Pharmacology Odense University Hospital Odense Denmark; ^4^ Centre for Public Health, Queen's University Belfast Belfast N Ireland

**Keywords:** clinical pharmacology, drug utilization, epidemiology pain, pharmacoepidemiology, prescribing

## Abstract

**Aims:**

Pregabalin and gabapentin are increasingly used for pain and other conditions. Concerns exist about overuse as well as potential misuse and abuse. To guide rational prescribing practices, we provide detailed nationwide data on the use of gabapentinoids in Denmark.

**Methods:**

We conducted a nationwide descriptive drug utilization study using the Danish healthcare registries to describe the use of gabapentinoids among Danish adults 2010–2023. We described overall use patterns, temporal trends, user characteristics, skewness of use, treatment duration and concomitant medication use.

**Results:**

The prevalence of gabapentinoid use increased almost four‐fold from 11 per 1000 adults in 2010 to 41 in 2023 with the highest use among individuals aged 80+ years (96 per 1000 in 2023). Gabapentin and pregabalin were used equally. The median age of users increased from 58 years in 2010 to 63 years in 2023, while a decline was observed in the proportion with concomitant use of other drug classes, including benzodiazepines or opioids. Only 7% of patients were continuously treated for 3 years following initiation, while 22% were currently treated after 3 years (allowing treatment breaks). The use of gabapentinoids were somewhat skewed with 1% of users accounting for 7.3% of use in 2023.

**Conclusion:**

The use of gabapentinoids has increased dramatically in recent years, in particular, among the elderly, and adherence was low. Increased attention to the increasing use of gabapentinoids is warranted to ensure rational use of this drug class.

**What is already known about this subject**

- The use of gabapentinoids continues to rise, partly attributed to shifts to nonopiate analgesics as well as increasing use for off‐label indications.
- Increasing use of gabapentinoids for off‐label indications with potentially limited clinical benefit may expose patients to potential adverse events, and the potential for misuse and abuse.
- Understanding current gabapentinoid prescribing practices is needed to help guide rational prescribing practices.

**What this study adds**

- Our study suggests an almost 4‐fold increase in gabapentinoid use in the Danish population from 2010 to 2023, most notably for the elderly, with almost 10% of those aged ≥80 years filling a gabapentinoid in 2023.
- Treatment durations were generally short, with about 1/5 continuing to receive gabapentinoid treatment 3 years after treatment initiation.
- Findings suggests that it is likely that pain conditions are a major factor explaining the increased use, most notably for gabapentin.

## 1 INTRODUCTION

Gabapentinoids, including pregabalin and gabapentin, are structural analogues for the neurotransmitter γ‐aminobutyric acid.^1^ Initially developed as anticonvulsants, gabapentinoids are also used for peripheral neuropathic pain, focal seizures and generalized anxiety disorder.

Recent years have seen a continued increase in use of gabapentinoids.^2^, ^3^, ^4^, ^5^ This has been attributed in part to concerns over opioid dependence and mortality,^2^ resulting in a hypothesized shift towards prescription of nonopiate analgesic agents. Increased prescribing has been further supported by widely adopted clinical guidelines (such as the UK National Institute for Health and Care Excellence guidelines), as well as off‐label use in a range of conditions such as alcohol addiction and bipolar disorder.^6^, ^7^ However, while modestly effective for neuropathic pain, in particular diabetic neuropathy and herpetic neuralgia for some patients,^8^ the evidence for gabapentinoids for a range of non‐neuropathic pain conditions and in acute pain management is limited.^9^, ^10^

Increasing use of gabapentinoids for off‐label indications, that may have limited clinical benefit, may expose patients to potential adverse events as well as the potential for misuse and abuse.^11^, ^12^ There have been increases in gabapentinoid dependence reported to the European Medicine Agency and gabapentinoid‐linked deaths.^13^, ^14^ Certain populations appear at higher risk of misuse, including those with a history of substance abuse, opioid users and users of antipsychotics or benzodiazepines.^3^, ^15^ However, the concomitant use of gabapentinoids with opioids and/or benzodiazepines appears common, raising concerns about the potential for adverse outcomes.^16^

Thus, in this study, we used nationwide Danish register‐based data to describe use of gabapentinoids over time, including the concomitant use of opioids and benzodiazepines, and the changing characteristics of users over time.

## 2 METHODS

In this nationwide descriptive drug utilization study, we used the Danish healthcare registries to describe the use of gabapentinoids in adults from 2010 to 2023.

### 2.1 Data sources

We used data from 3 nationwide registry sources. The National Prescription Registry contains individual level data on all prescription drugs filled by Danish residents since 1995.^17^ Data contain information on e.g. drug name, date of dispensing and quantity. The dosing information is not available, and the registry does not include information on drugs dispensed at hospitals. Drugs are categorized according to the Anatomic Therapeutic Chemical (ATC) index, a hierarchical classification system developed by the World Health Organization, and the quantity dispensed for each prescription is described by the number and strength of the pharmaceutical entities (e.g., tablets), as well as defined daily doses (DDD).^18^ Information on hospital diagnoses was obtained from the Danish National Patient Register^19^ and data on date of birth, death and immigration were obtained via the Danish Civil Registration System.^19^, ^20^ Linkage of the data sources was facilitated via a unique personal identification number (the CPR‐number), assigned to all Danish residents since 1968. All drug codes (ATC) and diagnoses codes (ICD‐10) are listed in Table S1.

### 2.2 Study population and study drugs

The study cohort included all individuals aged ≥18 years living in Denmark who were dispensed at least 1 prescription for a gabapentinoid, including pregabalin and gabapentin, between 1 January 2010 and 31 December 2023. Dispensed prescriptions for opioids and benzodiazepines were also identified to evaluate concomitant use patterns with gabapentinoids. Drugs were identified and categorized according to the ATC index and drug volume was expressed in DDDs, defined by the WHO as the assumed average maintenance dose per day for a drug used for its main indication in adults.^18^ One DDD correspond to 1800 mg of gabapentin or 300 mg of pregabalin. New users were defined as individuals with a gabapentinoid prescription with no prior prescription for gabapentinoids within the last 5 years.

### 2.3 Statistical analyses

#### 2.3.1 User characteristics

We described gabapentinoid users according to sex, age, comedications and morbidities in 2010, 2017 and 2023. Morbidities were assessed as hospital diagnoses recorded in the Patient Registry at any time prior to January 1 of the year being analysed, and comedications were those prescribed in the year prior to that being analysed. In addition, we described gabapentinoid users by indication and prescribers; categorized as general practice prescribers, hospital prescribers and others. Characteristics were described overall and for pregabalin and gabapentin users separately.

#### 2.3.2 Temporal trends

Several analyses were conducted to describe changes in gabapentinoid use over time. First, annual prevalence proportions of use of all gabapentinoids were calculated with all adults living in Denmark on 1 January each year included in the denominator. Second, annual incidence rates were calculated by dividing the annual number of new users by the follow‐up time in the total adult population of Denmark on the first day in each year. Third, the total drug use per year was displayed as the annual number of dispensed DDDs each year. Analyses were also conducted by sex and age categories (18–39, 40–59, 60–79 and ≥80 years) and for pregabalin and gabapentin separately.

#### 2.3.3 Inverse Lorenz curves and Gini coefficient

To assess whether there is a skewed use pattern among users of gabapentinoids, potentially indicating misuse, we generated inverse Lorenz curves on the total use of pregabalin and gabapentin for the years 2010, 2017 and 2023.^21^, ^22^ The Gini coefficient was calculated as a single measure with a value ranging from 0 to 1, with 0 denoting total equality and 1 denoting total inequality of use.^21^ The 1st, 10th, 50th and 90th percentiles were calculated for each curve. A high 1‐percentile would indicate that few users account for a high proportion of the total drug volume.

#### 2.3.4 Treatment duration and persistence

To evaluate duration of treatment, we used the *proportion of patients covered* method.^23^ For this, incident users were followed from the date of filling their first gabapentinoid prescription. On each day over the subsequent 3 years, we estimated the proportion of all gabapentinoid users still alive and not migrated that were covered by a gabapentinoid prescription on that day. An individual was considered to be covered by a prescription (*current user*) using a fixed prescription duration of 90 days.^23^ As such, an individual could be regarded as *dropped out of treatment* at 1 point in time and later be reclassified as *current user* upon filling a new prescription. Subgroup analyses were carried out according to age, sex and calendar year of first prescription. Second, treatment persistence was evaluated as a time‐to‐event variable using Kaplan–Meier survival curves where the first break in treatment (i.e. no new fill for 90 days) marked the event. As such, an individual was considered persistent until the first treatment break regardless of whether that individual reinitiated treatment later. In both approaches, users were censored at death, emigration and end of follow‐up (31 December 2023). The time between dispensings was also evaluated and the fixed period varied in a sensitivity analysis (from 90 to 180 days).

#### 2.3.5 Concomitant medication use

To evaluate concomitant use of opioids and benzodiazepines with gabapentinoids, we calculated the percentage of gabapentinoid users in each study year (at least 1 prescription fill in the given year), who used concurrent opioids and/or benzodiazepines. Concurrent use was defined as the dispensing of an opioid or a benzodiazepine within 90 days of (either before or after) a dispensed pregabalin or gabapentin prescription.

#### 2.3.6 Other

Stata Version 18 (StataCorp, College Station, TX, USA) was used for all analyses. The study was approved by the Danish Data Protection Agency. According to Danish law, studies based solely on register data do not require approval from an ethics review board.

### 2.4 Nomenclature of targets and ligands

Key protein targets and ligands in this article are hyperlinked to corresponding entries in http://www.guidetopharmacology.org, and are permanently archived in the Concise Guide to PHARMACOLOGY 2021/22 (Alexander *et al*., 2021).

## 3 RESULTS

Overall, we identified a total of 596 691 individuals filling at least 1 prescription for pregabalin or gabapentin within the study period (2010–2023), of whom 229 210 and 471 743 filled 1 or more prescriptions for pregabalin and gabapentin. Among gabapentin initiators, 4.2% had filled pregabalin within the last 5 years, while 30% of pregabalin initiators had filled gabapentin within the last 5 years. Throughout the study period, 31% filled only 1 prescription, 21% filled 2 or 3 prescriptions, while 48% filled > 3 prescriptions. As presented in Table 1, the median age of users in 2023 was 62 years (interquartile range 49–74), a slight increase from 2010 (58; interquartile range 45–70). Median age of gabapentin users was slightly higher than that for pregabalin (63 *vs*. 59 years in 2023; Table S2). Users of gabapentinoids were most often women (59 *vs*. 41%). Over the study period, the proportion of users with a history of certain comorbidities including essential hypertension, osteoarthritis and chronic pain increased. Pregabalin users were more likely to have certain comorbidities such as affective disorders (including depression) and chronic pain (Table S2). From 2010 there has been a decrease in the proportion of gabapentinoid users with coprescriptions for medication classes including antidepressants, benzodiazepines and opioids, most notably tramadol (Table 1). However, small increases in prescriptions for morphine were observed. Reductions in concomitant use of benzodiazepines or opioids were overall more notable for pregabalin than for gabapentin (Table S2).

**TABLE 1:** Characteristics of all gabapentinoid users in 2010, 2017 and 2023, defined as those filling a prescription during the respective year.

	2010	2017	2023
	(*n =* 47 361)	(*n =* 103 486)	(*n =* 194 278)
**Male %**	19 190 (41%)	41 887 (40%)	78 951 (41%)
**Age (median, IQR)**	58 (45–70)	59 (47–72)	62 (49–74)
**Comorbidities**			
Essential hypertension	9346 (20%)	27 120 (26%)	50 453 (26%)
Ischaemic heart disease	6063 (13%)	14 660 (14%)	25 923 (13%)
CKD	690 (1.5%)	2228 (2.2%)	4062 (2.1%)
Osteoarthritis	7324 (15%)	24 308 (23%)	52 653 (27%)
Diabetes	6784 (14%)	16 037 (15%)	35 048 (18%)
Affective disorders including depression	6483 (14%)	16 288 (16%)	27 597 (14%)
Depression	5951 (13%)	15 139 (15%)	25 545 (13%)
Migraine	852 (1.8%)	2827 (2.7%)	6681 (3.4%)
Cancer	5728 (12%)	14 524 (14%)	29 127 (15%)
COPD	3366 (7.1%)	8875 (8.6%)	14 621 (7.5%)
Chronic pain	4738 (10%)	16 823 (16%)	30 213 (16%)
Acute pain	267 (0.56%)	1631 (1.6%)	2680 (1.4%)
Fibromyalgia	184 (0.39%)	1217 (1.2%)	2768 (1.4%)
Rheumatoid arthritis	469 (0.99%)	1368 (1.3%)	2484 (1.3%)
**Comedications**			
Antipsychotics	6011 (13%)	11 083 (11%)	18 016 (9.3%)
Antidepressants	21 624 (46%)	37 948 (37%)	60 598 (31%)
Antiepileptic	3368 (7.1%)	5729 (5.5%)	9388 (4.8%)
Antidementia	294 (0.62%)	806 (0.78%)	1975 (1.0%)
Psychostimulants	716 (1.5%)	1951 (1.9%)	5062 (2.6%)
Anticholinergic	422 (0.89%)	595 (0.57%)	675 (0.35%)
Benzodiazepines	17 228 (36%)	24 795 (24%)	28 648 (15%)
***Opioids***	24 016 (51%)	50 511 (49%)	66 758 (34%)
Morphine	3123 (6.6%)	13 633 (13%)	26 899 (14%)
Oxycodone	7200 (15%)	9830 (9.5%)	17 403 (9.0%)
Tramadol	13 935 (29%)	30 834 (30%)	24 419 (13%)
Transdermal opioids	3615 (7.6%)	5491 (5.3%)	4761 (2.5%)
Codeine	5980 (13%)	8877 (8.6%)	11 090 (5.7%)
**Concomitant use with opioids or benzodiazepines**			
Any gabapentinoid	33 246 (70%)	66 616 (64%)	91 767 (47%)
Gabapentin	19 754 (42%)	46 549 (45%)	60 929 (31%)
Pregabalin	15 303 (32%)	23 679 (23%)	35 515 (18%)

Abbreviations: CKD, chronic kidney disease; COPD, chronic obstructive pulmonary disease; IQR, interquartile range.

Of the 8 873 567 gabapentinoid prescriptions identified, an indication was available for 7 189 257 prescriptions (81.02%). The proportion of available indication codes increased over time from 78.33 in 2015 to 86.12 in 2018 (electronic prescribing became mandatory in 2017) and 82.61% in 2023. We translated into English and grouped all indications with a frequency of >1000 prescriptions, corresponding to 12 unique indications covering 99.5% of prescriptions with a recorded indication. Translations and groupings are presented in Table S3. Trends in prescribing by indication are presented in Figure S1. Overall, the most common indication for gabapentinoid prescriptions was for pain, particularly for gabapentin for which by 2023 84% of prescriptions indicated for pain. For pregabalin, 30.5% of prescriptions in 2023 were indicated for anxiety.

### 3.1 Temporal trends

**FIGURE 1:**
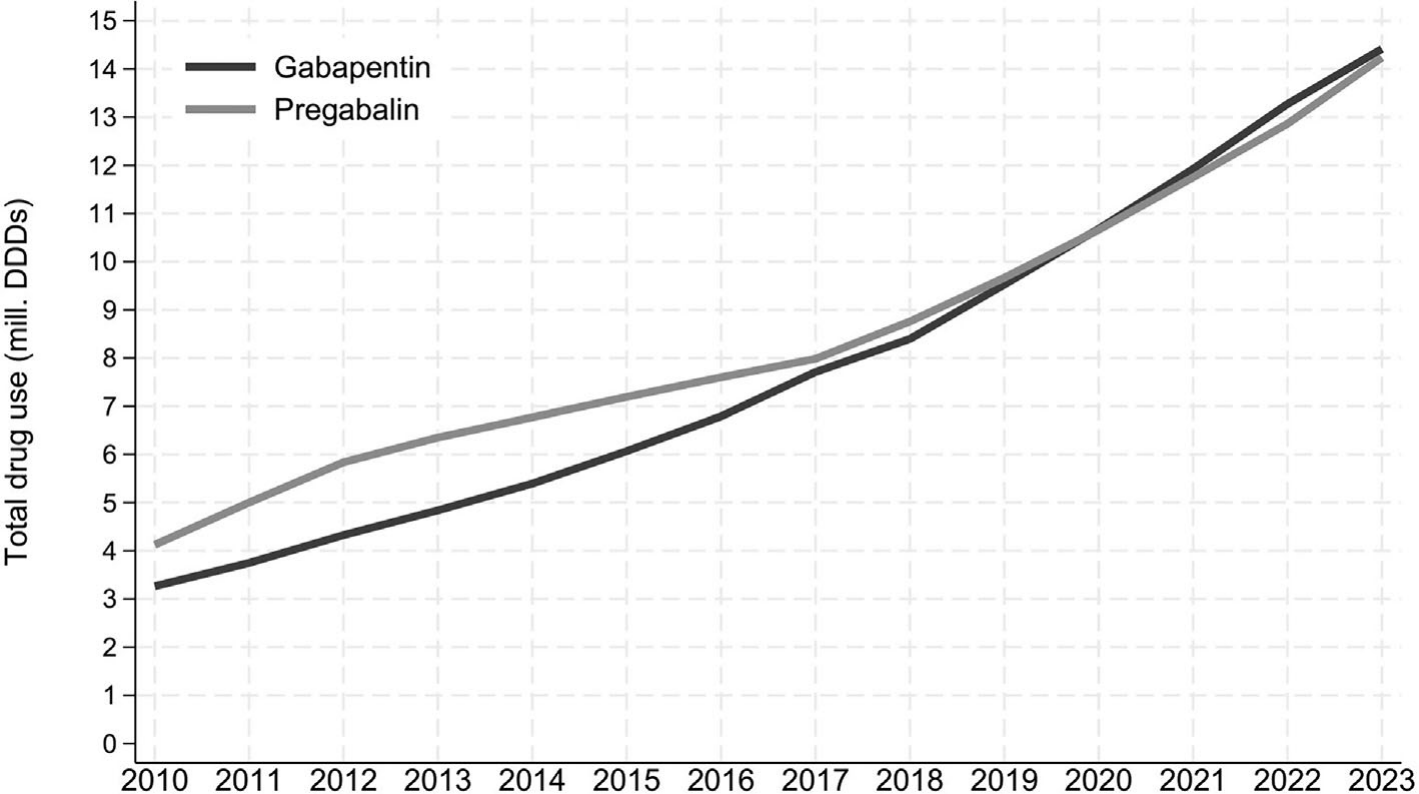
Total annual amount of pregabalin and gabapentin used among Danish adults 2010 to 2023, expressed as the total amount of dispensed defined daily doses (DDDs) at community pharmacies. One DDD corresponds to 1800 mg of gabapentin or 300 mg of pregabalin.

Over the study period, the total use of all gabapentinoids increased gradually to a total of 29 million DDDs dispensed in 2023 (Figure S2). Total use of pregabalin was slightly higher than for gabapentin until 2017 (Figure 1). Thereafter, both drugs were used to a similar extent. We observed an increase in the annual prevalence of gabapentinoid use from 11 per 1000 adult individuals in 2010 to 41 per 1000 in 2023 (Figure S3). This was driven by an increase in new users (incidence rate) of all gabapentinoids from 5.0 incident users per 1000 person‐years in 2010 to 15 per 1000 person‐years in 2023 (Figure  S4). Similar trends were observed for gabapentin (Figure S5), while incidence rates for new pregabalin use remained relatively stable throughout the study period (Figure S6).

**FIGURE 2:**
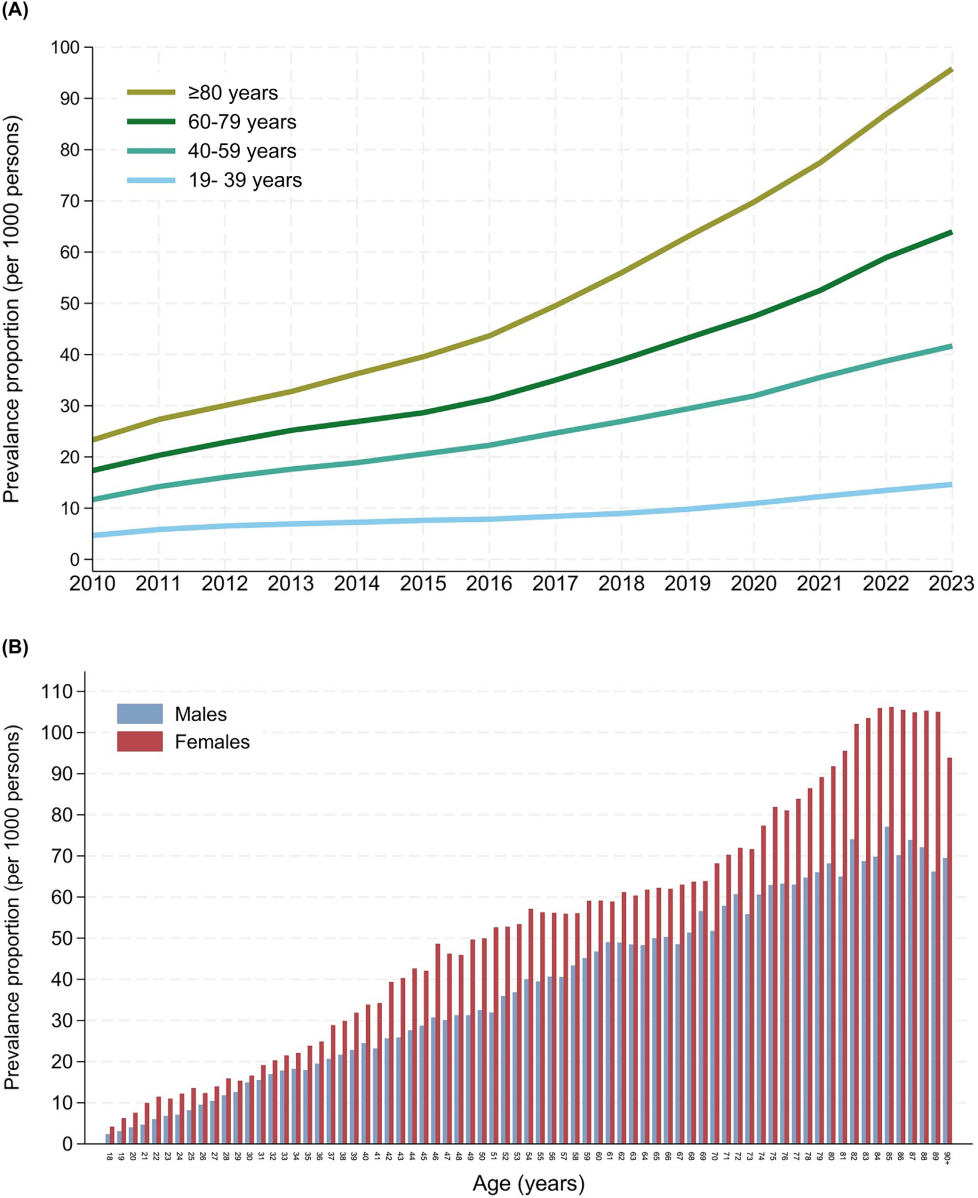
Trends in gabapentinoid prescribing by age category. (A) The annual prevalence proportion of gabapentinoid users per 1000 individuals from 2010 to 2023, by age group. (B) Prevalence proportion per 1000 persons of all gabapentinoids by age and sex in 2023.

The prevalence increased in both sexes over the study period and was higher among women than men (Figure S7; 48/1000 women vs 34/1000 men in 2023). The prevalence of use increased with age throughout the study period (Figure 2A) and in 2023 (Figure 2B), women showed a peak prevalence of 106/1000 at age 85 years, compared to a peak prevalence of 77/1000 for men aged 85 years. These age and sex differences have increased over time, as illustrated by the corresponding figures for 2010 (Figure S8) and 2017 (Figure S9).

### 3.2 Prescriber responsibility

**FIGURE 3:**
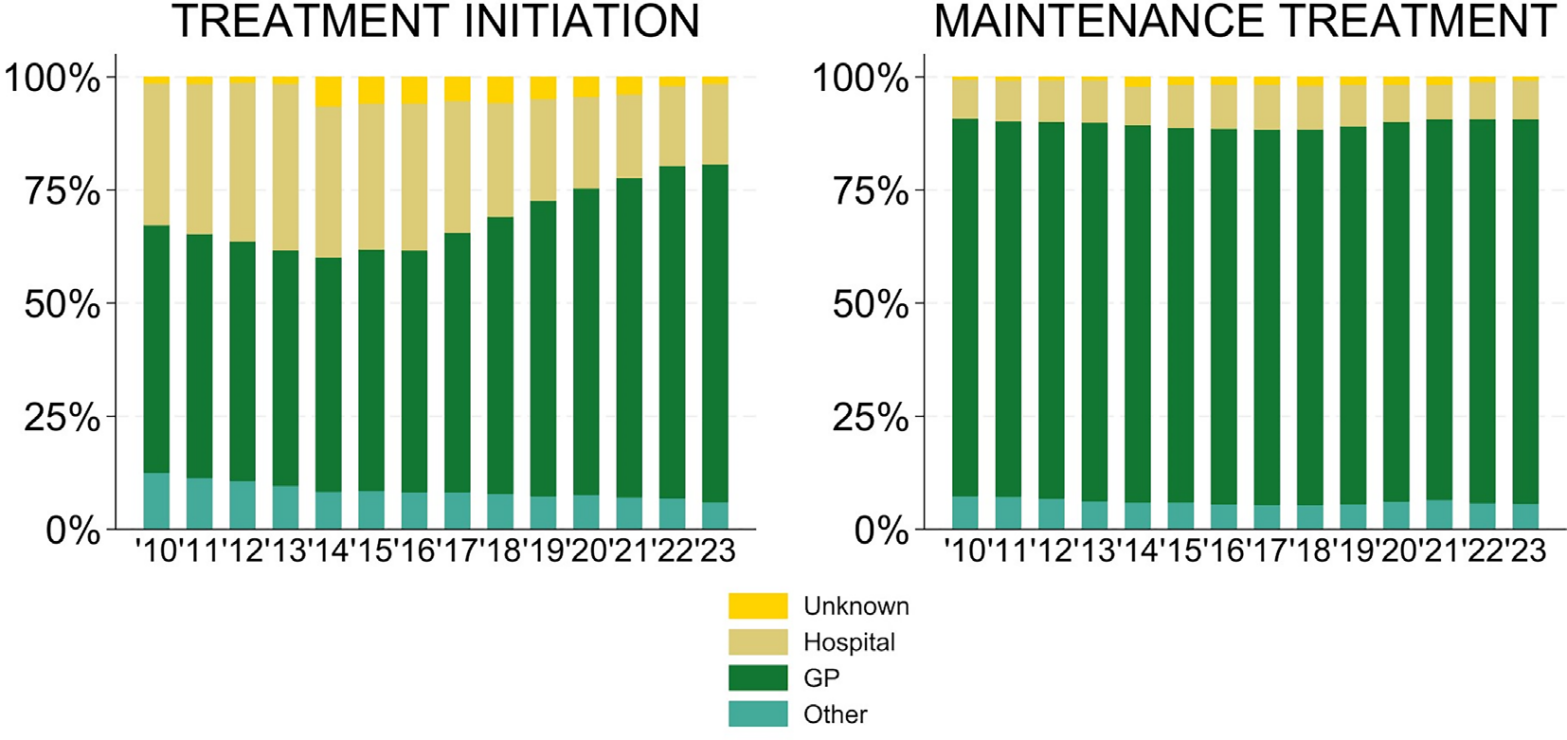
Proportion of all gabapentinoid prescriptions initiated and maintained by speciality of prescribers (hospital prescribers, general practitioners and other) from 2010 to 2023.

Overall, the majority of gabapentinoid prescriptions are initiated in general practice with 75% initiated in general practice in 2023 (Figure 3). This proportion has increased over time while the proportion of prescriptions initiated in secondary care has decreased. This shift was more pronounced for gabapentin, whereas pregabalin was more often initiated by hospital prescribers and with less variation over time (25% in 2023; Figure S10). The proportion of prescriptions issued by general practitioners were slightly higher among older compared to younger patients (data not shown).

### 3.3 Treatment duration

**FIGURE 4:**
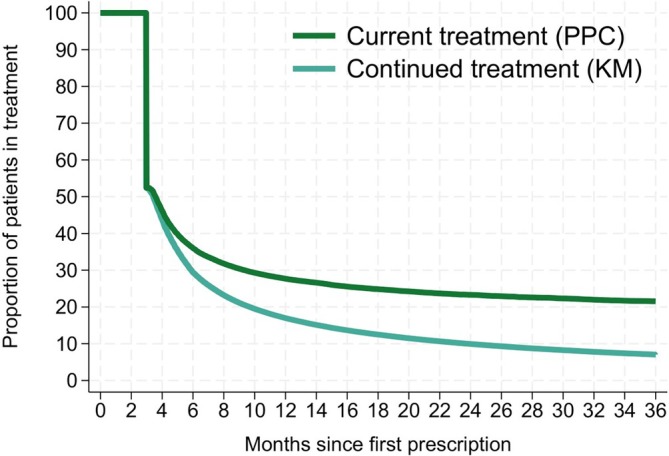
Duration of gabapentinoid therapy calculated as the proportion of patients in current treatment (proportion of patients covered; PPC) and in continuous treatment (Kaplan–Meier; KM).

As illustrated in Figure 4, 3 years after gabapentinoid initiation, 7% were still in continuous treatment, i.e. had not had a treatment break (Kaplan–Meier survival), while 22% of patients still alive were currently being treated (*proportion of patients covered*). This was somewhat dependent on age, with a slightly greater proportion of those aged ≥80 years remaining alive and on current treatment after 3 years (27%; Figure S11). No differences were observed by sex, nor were there major differences when assuming a 180‐day prescription duration instead of 90 days (data not shown).

### 3.4 Skewness in use

**FIGURE 5:**
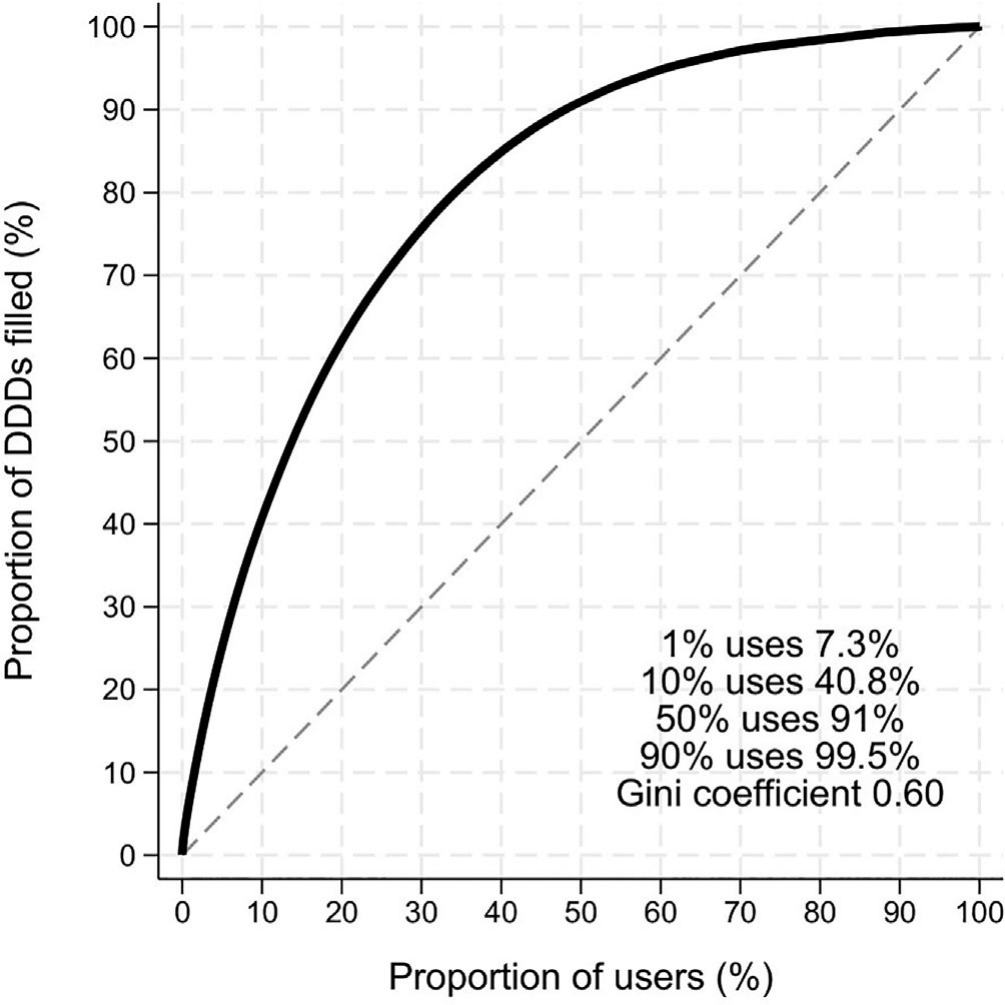
Inverse Lorenz curve of gabapentinoid use. The curve denotes the proportion of the total filled amount of gabapentinoids (measured in defined daily doses [DDDs]) in 2023 that is accounted for by which proportion of all users ranked from the highest to the lowest users.

The inverse Lorenz curve for all gabapentinoids in 2023 is shown in Figure 5. The curve was moderately skewed, with 1% of the highest users representing 7.3% of the total amount used and 50% of users representing 91% of the total amount of gabapentinoids filled. No differences over time nor by individual gabapentinoid were observed (data not shown).

## 4 DISCUSSION

We provide detailed analyses on the use of gabapentinoids in Denmark, documenting an almost 4‐fold increase from 2010 to 2023, with 4.1% of the entire adult Danish population filling a prescription for gabapentinoids in 2023. Of note, these increases were most pronounced among the older population with almost 10% of those aged ≥80 years filling a gabapentinoid in 2023. Treatment durations were generally short, with about 1/5 continuing to receive gabapentinoid treatment 3 years after treatment initiation and use was moderately skewed with 1% of users filling 7.3% of all gabapentinoids.

The main strength of the present study is the nationwide data capture, allowing assessment of the entire population's use of gabapentinoids, eliminating selection or sampling bias. Several weaknesses also need to be acknowledged. Importantly, while comprehensive, with the ability to evaluate indication for gabapentinoid use, this was missing for almost 20% of prescriptions, which therefore limited the evaluation of how findings reflected rational use and how treatment patterns adhered to guidelines. Similarly, our findings of high levels of short‐term use and poor adherence could not be further scrutinized as we were unable to determine if treatment was intended to be short term or stopped due to lack of efficacy, side effects or improvement of the underlying condition. Finally, our data covered community pharmacy fills and thus could not account for in‐hospital use of gabapentinoids; however, this only corresponded to 1.5% of the total Danish use of gabapentinoids in 2023.^24^

The almost 4‐fold increase in the use of gabapentinoid use from 2010 to 2023 constitutes the main finding of the present study. The indication data available suggest that it is likely that pain conditions are a major factor explaining the increased use,^10^, ^25^ most notably for gabapentin. Over the same period, a key focus from health organizations globally, including in Denmark, has seen the reduction of opioid use. Indeed, the use of opioids, most notably tramadol, has been reduced particularly since Danish legislative changes in 2018,^26^ which coincides with a seemingly accelerated growth in the use of gabapentinoids. While further elucidation of the current results is needed, it may be that initiatives to reduce opioids might inadvertently increase the use of gabapentinoids. Considering the somewhat limited evidence for use of gabapentinoids for pain,^9^, ^10^ this might not reflect rational use and therefore the risk of channelling patients from opioid to gabapentinoid use may need to be considered in initiatives aimed at reduced prescribing of opioids.

Given the documented tolerability of these medications, the very low adherence to gabapentinoid treatment reported in this study constitutes an area where additional investigations are needed, in particular of the high proportion of users only filling a single prescription (i.e., early discontinuation). While the present study does not provide data as to the reasons for this finding, there are several potential explanations. It is possible that this may reflect an increase in gabapentinoids for postoperative pain management, as a switch from opioid use, for which single prescriptions are indicated,^10^ Additionally, the large proportion ceasing treatment early raises the hypothesis that gabapentinoids are used outside robust indications. In such settings, patients are less likely to tolerate side effects, and their resulting restrictions such as on the ability to drive under the influence of gabapentinoids, which is illegal in Denmark,^26^ and thus resulting in reduced compliance. Of note, one important finding of this study was the increase and particularly high use of gabapentinoids among the elderly. While also probably reflecting pain management, gabapentin is also used to treat behavioural and psychological symptoms of dementia. Given this finding, the lower adherence to gabapentinoids in this study may not be surprising, as older patients who have more polypharmacy, greater comorbidities and decreased renal function, are at increased risk for adverse events.

The potential for abuse and misuse of gabapentinoids has been heavily debated.^11^, ^27^ In the present study, we observe a moderately skewed use with 1% of users filling 7.3% of all gabapentinoids, which is lower than for e.g. opioids (1% of users account for 18% of total use^28^) and sumatriptan (1% of users account for 20% of total use^29^). Of note, in situations of various indications with variable dosages and importantly, with a large proportion of gabapentinoid users that stop treatment early, measures of skewness will be inflated. Those filling 1 prescription will fill less drug than those continuously treated, irrespective of those continuously treated receiving normal doses of gabapentinoids. As such, we do not find evidence of major skewness in the overall use of gabapentinoids and thus do not observe signs of abuse of this drug class. However, considering the high‐level nationwide approach to our data analysis, our data should not be interpreted as proving that no such abuse or misuse can exist on the level of the single patient.

In summary, we have documented a marked increase in the use of gabapentinoids in Denmark over the last decade. This increase is particularly pronounced among the elderly. Further, the overall adherence to gabapentinoid treatment appears limited, and use is moderately skewed. Therefore, moving forward, prioritizing the rationale use of gabapentinoids is crucial for advancing rational pharmacotherapy efforts.

## AUTHOR CONTRIBUTIONS

B.H. and A.P. conceptualized the study. All authors contributed to study design. A.P. and B.H. supervised the study and were responsible for data acquisition. M.O. performed the statistical analysis. All authors took part in data analysis and interpretation. A.P. and B.H. drafted the manuscript. All authors participated in manuscript review and editing, agreed on the content of the manuscript, and approved the final version.

## CONFLICT OF INTEREST STATEMENT

A.P. reports participation in research projects funded by Alcon, Almirall, Astellas, AstraZeneca, Boehringer‐Ingelheim, Novo Nordisk, Servier and LEO Pharma, all regulator‐mandated phase IV‐studies, all with funds paid to the institution where he was employed (no personal fees) and with no relation to the work reported in this paper. J.K. has received honoraria from Eisai on projects not related to this paper. The remaining authors have no conflicts of interest to declare.

## Supporting information


**TABLE S1** Codes and definitions.
**TABLE S2** Characteristics of pregabalin and gabapentin users in 2010, 2017 and 2023, defined as those filling at least 1 gabapentinoid prescription during the respective year.
**TABLE S3** Translations and grouping of indications for gabapentinoids.
**FIGURE S1** Proportion of gabapentinoids (overall and specified as gabapentin and pregabalin) by recorded indication 2010–2023. Turquoise denotes anxiety, dark green pain, blue epilepsy and beige missing.
**FIGURE S2** Total amount of gabapentinoids used per year among Danish adults, expressed as the total amount of dispensed defined daily doses (DDDs) at community pharmacies. One DDD corresponds to 1800 mg of gabapentin or 300 mg of pregabalin.
**FIGURE S3** The prevalence proportion per 1000 persons of all gabapentinoids during 2010–2023. Prevalent use was defined as a fill for a gabapentinoids within the corresponding year.
**FIGURE S4** Incidence rates of new users of gabapentinoids during 2010–2023. New (incident) use was defined as the first fill for a gabapentinoid in at least 5 years.
**FIGURE S5** Annual incidence rates of new users of gabapentin during 2010–2023. New (incident) use was defined as the first fill of a gabapentinoid in at least 5 years. New (incident) use of gabapentin with recent use of pregabalin (within 5 years) was not included as new (incident) gabapentin use.
**FIGURE S6** Annual incidence rates of new users of pregabalin during 2010–2023. New (incident) use was defined as the first fill of pregabalin in at least 5 years. New (incident) use of pregabalin with recent use of gabapentin (within 5 years) was not included as new (incident) pregabalin use.
**FIGURE S7** The prevalence proportion per 1000 persons per year of all gabapentinoids, by sex, during 2010–2023. Prevalent use was defined as a fill for a gabapentinoids within the corresponding year.
**FIGURE S8** The prevalence proportion per 1000 persons of gabapentinoids by age and sex during 2010.
**FIGURE S9** The prevalence proportion per 1000 persons of gabapentinoids by age and sex during 2017.
**FIGURE S10** Proportion of gabapentinoids (overall and specified as gabapentin and pregabalin) that were initiated (defined as first prescription in 5 years for the given patient) and maintained (defined as all other prescriptions) speciality of prescribers annually during 2010–2023. Turquoise denotes private practicing specialist prescribers, dark green denotes general practitioners, beige denotes hospital prescribers and yellow denotes unknown prescribers.
**FIGURE S11** Duration of gabapentinoid therapy specified as the proportion of patients covered (current treatment) since each patient's incident (first in at least 5 years) prescription during 2010–2023, specified by age strata.

## Data Availability

Data cannot be shared publicly due to legal restrictions. Access to data can be requested through the Danish Registers and Statistics Denmark.

## References

[1] Patel R , Dickenson AH . Mechanisms of the gabapentinoids and α 2 δ‐1 calcium channel subunit in neuropathic pain. Pharmacol Res Perspect. 2016;4(2):e00205. doi:10.1002/prp2.205 27069626 PMC4804325

[2] Goodman CW , Brett AS . Gabapentin and pregabalin for pain ‐ is increased prescribing a cause for concern? N Engl J Med. 2017;377(5):411‐414. doi:10.1056/NEJMp1704633 28767350

[3] Schjerning O , Pottegård A , Damkier P , Rosenzweig M , Nielsen J . Use of pregabalin – a Nationwide Pharmacoepidemiological drug utilization study with focus on abuse potential. Pharmacopsychiatry. 2016;49(04):155‐161. doi:10.1055/s-0042-101868 26951495

[4] Torrance N , Veluchamy A , Zhou Y , et al. Trends in gabapentinoid prescribing, co‐prescribing of opioids and benzodiazepines, and associated deaths in Scotland. Br J Anaesth. 2020;125(2):159‐167. doi:10.1016/j.bja.2020.05.017 32571568

[5] Xia Y , Forget P . Opioid and gabapentinoid prescriptions in England from 2015 to 2020. PLoS ONE. 2022;17(11):e0276867. doi:10.1371/journal.pone.0276867 36441772 PMC9704625

[6] Goodman CW , Brett AS . A clinical overview of off‐label use of Gabapentinoid drugs. JAMA Intern Med. 2019;179(5):695. doi:10.1001/jamainternmed.2019.0086 30907944

[7] Peckham AM , Evoy KE , Ochs L , Covvey JR . Gabapentin for off‐label use: evidence‐based or cause for concern? Subst Abus Res Treat. 2018;12:117822181880131. doi:10.1177/1178221818801311 PMC6153543 30262984

[8] Williams CD , Al‐Jammali Z , Herink MC . Gabapentinoids for pain: a review of published comparative effectiveness trials and data submitted to the FDA for approval. Drugs. 2023;83(1):37‐53. doi:10.1007/s40265-022-01810-3 36529848

[9] Goodman CW , Brett AS . Gabapentinoids for pain: potential unintended consequences. Am Fam Physician. 2019;100:672‐675. 31790179

[10] Verret M , Lauzier F , Zarychanski R , et al. Perioperative use of Gabapentinoids for the Management of Postoperative Acute Pain. Anesthesiology. 2020;133(2):265‐279. doi:10.1097/ALN.0000000000003428 32667154

[11] Evoy KE , Sadrameli S , Contreras J , Covvey JR , Peckham AM , Morrison MD . Abuse and misuse of pregabalin and gabapentin: a systematic review update. Drugs. 2021;81(1):125‐156. doi:10.1007/s40265-020-01432-7 33215352

[12] Hägg S , Jönsson AK , Ahlner J . Current evidence on abuse and misuse of Gabapentinoids. Drug Saf. 2020;43(12):1235‐1254. doi:10.1007/s40264-020-00985-6 32857333 PMC7686181

[13] Chiappini S , Schifano F . A decade of Gabapentinoid misuse: an analysis of the European medicines Agency's “suspected adverse drug reactions” database. CNS Drugs. 2016;30(7):647‐654. doi:10.1007/s40263-016-0359-y 27312320

[14] Kriikku P , Ojanperä I . Pregabalin and gabapentin in non‐opioid poisoning deaths. Forensic Sci Int. 2021;324:110830. doi:10.1016/j.forsciint.2021.110830 34000615

[15] Smith RV , Havens JR , Walsh SL . Gabapentin misuse, abuse and diversion: a systematic review. Addiction. 2016;111(7):1160‐1174. doi:10.1111/add.13324 27265421 PMC5573873

[16] Ashworth J , Bajpai R , Muller S , et al. Trends in gabapentinoid prescribing in UK primary care using the clinical practice research datalink: an observational study. Lancet reg Heal ‐ Eur. 2023;27:100579. doi:10.1016/j.lanepe.2022.100579 PMC10105252 37069852

[17] Wallach Kildemoes H , Toft Sørensen H , Hallas J . The Danish National Prescription Registry. Scand J Public Health. 2011;39(7_suppl):38‐41. doi:10.1177/1403494810394717 21775349

[18] WHOCC ‐ ATC/DDD Index. n.d. Accessed December 15, 2023. Available: https://www.whocc.no/atc_ddd_index_and_guidelines/atc_ddd_index/

[19] Schmidt M , Schmidt SAJ , Sandegaard JL , Ehrenstein V , Pedersen L , Sørensen HT . The Danish National Patient Registry: a review of content, data quality, and research potential. Clin Epidemiol. 2015;7:449‐490. doi:10.2147/CLEP.S91125 26604824 PMC4655913

[20] Pedersen CB . The Danish civil registration system. Scand J Public Health. 2011;39(7_suppl):22‐25. doi:10.1177/1403494810387965 21775345

[21] Hallas J , Stovring H . Templates for analysis of individual‐level prescription data. Toxicology. 2006;98:260‐265. 10.1111/j.1742-7843.2006.pto_257.x 16611200

[22] Rasmussen L , Wettermark B , Steinke D , Pottegård A . Core concepts in pharmacoepidemiology: measures of drug utilization based on individual‐level drug dispensing data. Pharmacoepidemiol Drug Saf. 2022;31(10):1015‐1026. doi:10.1002/pds.5490 35819240 PMC9545237

[23] Rasmussen L , Pratt N , Hansen MR , Hallas J , Pottegård A . Using the “proportion of patients covered” and the Kaplan‐Meier survival analysis to describe treatment persistence. Pharmacoepidemiol Drug Saf. 2018;27(8):867‐871. doi:10.1002/pds.4582 29952045

[24] Sundhedsdatastyrelsen ‐ Statistikker. n.d. Accessed February 6, 2023. Available: https://medstat.dk/en

[25] Huang LL , Wright JA , Fischer KM , et al. Gabapentinoid prescribing practices at a large Academic Medical Center. Mayo Clin Proc Innov Qual Outcomes. 2023;7(1):58‐68. doi:10.1016/j.mayocpiqo.2022.12.002 36660177 PMC9842797

[26] Danish Medicines Agency . Ny udleveringsstatus for visse opioider træder i kraft den 1. januar 2018. Accessed April 26, 2023. https://laegemiddelstyrelsen.dk/da/nyheder/2017/ny-udleveringsstatus-for-visse-opioider-traeder-i-kraft-den-1-januar-2018/

[27] Pottegård A , Tjäderborn M , Schjerning O , Nielsen J , Damkier P , Bodén R . Re: pregabalin prescriptions in the United Kingdom ‐ a drug utilisation study of the health improvement network (THIN) primary care database by Asomaning et al. Int J Clin Pract. 2016;70(8):696. doi:10.1111/ijcp.12836 27466015

[28] Hallas J . Drug utilization statistics for individual‐level pharmacy dispensing data. Pharmacoepidemiol Drug Saf. 2005;14(7):455‐463. 15651088 10.1002/pds.1063

[29] Gaist D , Sindrup S , Hallas J , Gram LF . Misuse of sumatriptan. Lancet. 1994;344(8929):1090. doi:10.1016/S0140-6736(94)91746-9 7934471

