# Supplementary material for: Trends in gabapentinoid prescribing: A nationwide Danish drug utilization study
Source: Br J Clin Pharmacol. 2025 Apr 9;91(9):2515–23. doi: 10.1002/bcp.70060 (PMC12381606; doi:10.1002/bcp.70060)

Trends in gabapentinoid prescribing: a nationwide Danish drug utilization study

Pottegård et al.

SUPPLEMENTARY MATERIAL

Table of Contents

**Table S1**	Codes and definitions	Page 3
**Table S2**	Characteristics of pregabalin and gabapentin users in 2010, 2017 and 2023, defined as those filling at least one gabapentinoid prescription during the respective year.	Page 4
**Table S3**	Translations and grouping of Indications for gabapentinoids	Page 5
**Figure S1**	Proportion of gabapentinoids (overall and specified as gabapentin and pregabalin) by recorded indication 2010 to 2023.	Page 6
**Figure S2**	Total amount of gabapentinoids used per year among Danish adults, expressed as the total amount of dispensed defined daily doses (DDDs) at community pharmacies.	Page 7
**Figure S3**	The prevalence proportion per 1,000 persons of all gabapentinoids during 2010 to 2023.	Page 8
**Figure S4**	Incidence rates of new users of gabapentinoids during 2010 to 2023.	Page 9
**Figure S5**	Annual incidence rates of new users of gabapentin during 2010 to 2023.	Page 10
**Figure S6**	Annual incidence rates of new users of pregabalin during 2010 to 2023.	Page 11
**Figure S7**	The prevalence proportion per 1,000 persons per year of all gabapentinoids, by sex, during 2010 to 2023.	Page 12
**Figure S8**	The prevalence proportion per 1,000 persons of gabapentinoids by age and sex during 2010.	Page 13
**Figure S9**	The prevalence proportion per 1,000 persons of gabapentinoids by age and sex during 2017.	Page 14
**Figure S10**	Proportion of gabapentinoids (overall and specified as gabapentin and pregabalin) that were initiated (defined as first prescription in five years for the given patient) and maintained (defined as all other prescriptions) speciality of prescribers annually during 2010 to 2023.	Page 15
**Figure S11**	Duration of gabapentinoid therapy specified as the proportion of patients covered (current treatment) since each patient's incident (first in at least five years) prescription during 2010 to 2023, specified by age strata.	Page 16

eTable 1 Codes and definitions

Drug	ATC code
Gabapentinoids	
Gabapentin	N03AX12/N02BF01 [a]
Pregabalin	N03AX16/N02BF02 [a]
Opioids	N02A*, R05DA04, N02AJ06, N02AJ07, N02BA75
Antidementia drugs	N06D
Benzodiazepines	N05BA, N05CD, N05CF
Antipsychotics	N05A* (except N05AN01)
Antidepressants	N06A*
Antiepileptic drugs	N03* (except N03AE01)
Psychostimulants	N06B*
Anticholinergic	N04A*

Comorbidity	ICD-10 code
Essential hypertension	I10
Ischemic heart disease	I20-25
Chronic kidney disease	N18 and N118
Osteoarthritis	M15-M19
Diabetes	E10-E14, (prescriptions: A10)
Affective disorders excluding depression	F30-F31 and F34-39
Depression	F32-F33
Migraine	G43
Cancer (excluding nonmelanoma skin cancer)	C00-C98 (excluding C44)
Mental and behavioral disorders due to psychoactive substance use	F10-19
Chronic obstructive pulmonary disease	J42-44
Pain	R52, R529
Acute	R520
Chronic	R522A, R522
Fibromyalgia	M797
Rheumatoid arthritis	M05

[a] ATC code change. N02BF* applying from 2022

Table S2 Characteristics of pregabalin and gabapentin users in 2010, 2017 and 2023, defined as those filling at least one gabapentinoid prescription during the respective year.

	2010		2017		2023	
	**Pregabalin** (n=20,548)	**Gabapentin** (n=26,813)	**Pregabalin** (n=33,680)	**Gabapentin** (n=69,806)	**Pregabalin** (n=66,400)	**Gabapentin** (n=127,878)
**Male %**	7829 (38%)	11,361 (42%)	13,190 (39%)	28,697 (41%)	25,899 (39%)	53,052 (41%)
**Age (median, IQR)**	54 (42-67)	62 (49-72)	56 (44-69)	61 (49-73)	59 (45-73)	63 (51-75)
**Comorbidities**						
Essential hypertension	3300 (16%)	6046 (23%)	7714 (23%)	19,406 (28%)	16,001 (24%)	34,452 (27%)
Ischemic heart disease	2004 (9.8%)	4059 (15%)	4055 (12%)	10,605 (15%)	8079 (12%)	17,844 (14%)
CKD	206 (1.0%)	484 (1.8%)	677 (2.0%)	1551 (2.2%)	1444 (2.2%)	2618 (2.0%)
Osteoarthritis	2599 (13%)	4725 (18%)	6531 (19%)	17,777 (25%)	16,033 (24%)	36,620 (29%)
Diabetes	2252 (11%)	4532 (17%)	4637 (14%)	11,400 (16%)	11,377 (17%)	23,671 (19%)
Affective disorders including depression	4390 (21%)	2093 (7.8%)	9374 (28%)	6914 (10%)	15,030 (23%)	12,567 (9.8%)
Depression	4019 (20%)	1932 (7.2%)	8663 (26%)	6476 (9.3%)	13,869 (21%)	11,676 (9.1%)
Migraine	410 (2.0%)	442 (1.6%)	1012 (3.0%)	1815 (2.6%)	2409 (3.6%)	4272 (3.3%)
Cancer	2187 (11%)	3541 (13%)	4583 (14%)	9941 (14%)	9870 (15%)	19,257 (15%)
COPD	1191 (5.8%)	2175 (8.1%)	2811 (8.3%)	6064 (8.7%)	5176 (7.8%)	9445 (7.4%)
Chronic Pain	2230 (11%)	2508 (9.4%)	6907 (21%)	9916 (14%)	12,969 (20%)	17,244 (13%)
Acute Pain	101 (0.49%)	166 (0.62%)	520 (1.5%)	1111 (1.6%)	962 (1.4%)	1718 (1.3%)
Fibromyalgia	109 (0.53%)	75 (0.28%)	512 (1.5%)	705 (1.0%)	1183 (1.8%)	1585 (1.2%)
Rheumatoid arthritis	157 (0.76%)	312 (1.2%)	326 (0.97%)	1042 (1.5%)	747 (1.1%)	1737 (1.4%)
**Co-medications**						
Antipsychotics	4463 (22%)	1548 (5.8%)	7470 (22%)	3613 (5.2%)	11,475 (17%)	6541 (5.1%)
Antidepressants	11,999 (58%)	9625 (36%)	18,564 (55%)	19,384 (28%)	29,992 (45%)	30,606 (24%)
Antiepileptic	1815 (8.8%)	1553 (5.8%)	2987 (8.9%)	2742 (3.9%)	4663 (7.0%)	4725 (3.7%)
Anti-dementia	128 (0.62%)	166 (0.62%)	366 (1.1%)	440 (0.63%)	910 (1.4%)	1065 (0.83%)
Psychostimulants	502 (2.4%)	214 (0.80%)	1175 (3.5%)	776 (1.1%)	2936 (4.4%)	2126 (1.7%)
Anticholinergic	323 (1.6%)	99 (0.37%)	441 (1.3%)	154 (0.22%)	437 (0.66%)	238 (0.19%)
Benzodiazepines	8744 (43%)	8484 (32%)	10,835 (32%)	13,960 (20%)	13,422 (20%)	15,226 (12%)
Opioids	9346 (45%)	14,670 (55%)	15,017 (45%)	35,494 (51%)	22,734 (34%)	44,024 (34%)
Morphine	1096 (5.3%)	2027 (7.6%)	4125 (12%)	9508 (14%)	9357 (14%)	17,542 (14%)
Oxycodone	2699 (13%)	4501 (17%)	3108 (9.2%)	6722 (9.6%)	5974 (9.0%)	11,429 (8.9%)
Tramadol	5342 (26%)	8593 (32%)	8520 (25%)	22,314 (32%)	7908 (12%)	16,511 (13%)
Transdermal opioids	1508 (7.3%)	2107 (7.9%)	2099 (6.2%)	3392 (4.9%)	2066 (3.1%)	2695 (2.1%)
Codeine	2428 (12%)	3552 (13%)	2713 (8.1%)	6164 (8.8%)	3750 (5.6%)	7340 (5.7%)
**Concomitant use with opioids or benzodiazepines**						
Any gabapentinoid	13,991 (68%)	19,255 (72%)	20,852 (62%)	45,764 (66%)	32,148 (48%)	59,619 (47%)
Gabapentin	565 (2.7%)	19,189 (72%)	908 (2.7%)	45,641 (65%)	1574 (2.4%)	59,355 (46%)
Pregabalin	13,970 (68%)	1333 (5%)	20,821 (62%)	2858 (4.1%)	32,048 (48%)	3467 (2.7%)

Table S3 Translations and grouping of Indications for gabapentinoids

Indication (Danish)	Translation	Indication group
mod smerter	Against pain	Pain
mod neuropatiske smerter	Against neuropathic pain	Pain
mod angst	against anxiety	Anxiety
mod epilepsy	Against epilepsy	Epilepsy
mod perifere neuropatiske smerter	Against peripheral neuropathic pain	Pain
mod kvalme	Against nausea	Other/Unclassified
mod stærke smerter	Against severe pain	Pain
mod tremor	Against tremor	Other/Unclassified
ved trigeminusneuralgi	Against trigeminal neuralgia	Pain
smertestillende	Analgesic	Pain
Restless Legs Syndrome (RLS)	Restless Legs Syndrome (RLS)	Other/Unclassified
mod neuropatiske smerter ved cancer	Against neuropathic pain in cancer	Pain

Figure S1

Proportion of gabapentinoids (overall and specified as gabapentin and pregabalin) by recorded indication 2010 to 2023. Turquoise denotes anxiety, dark green denotes pain, blue denoted epilepsy and beige denotes missing.

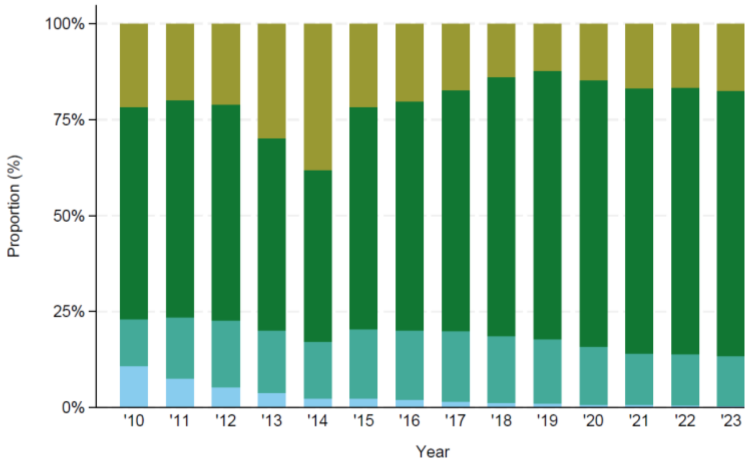

A. All gabapentinoids

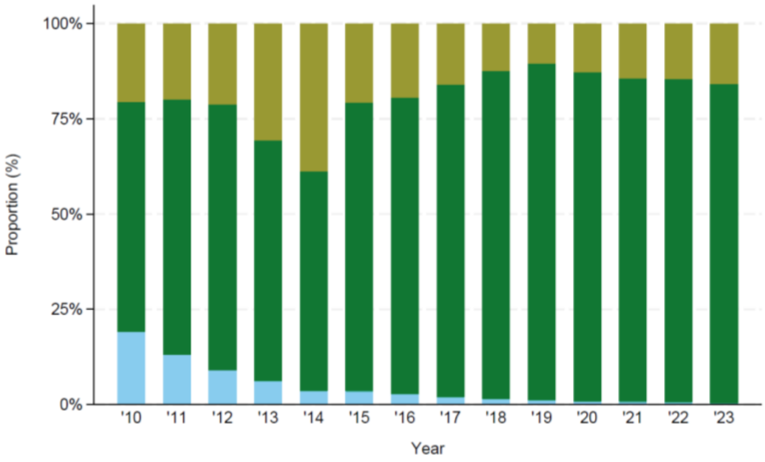

B. Gabapentin

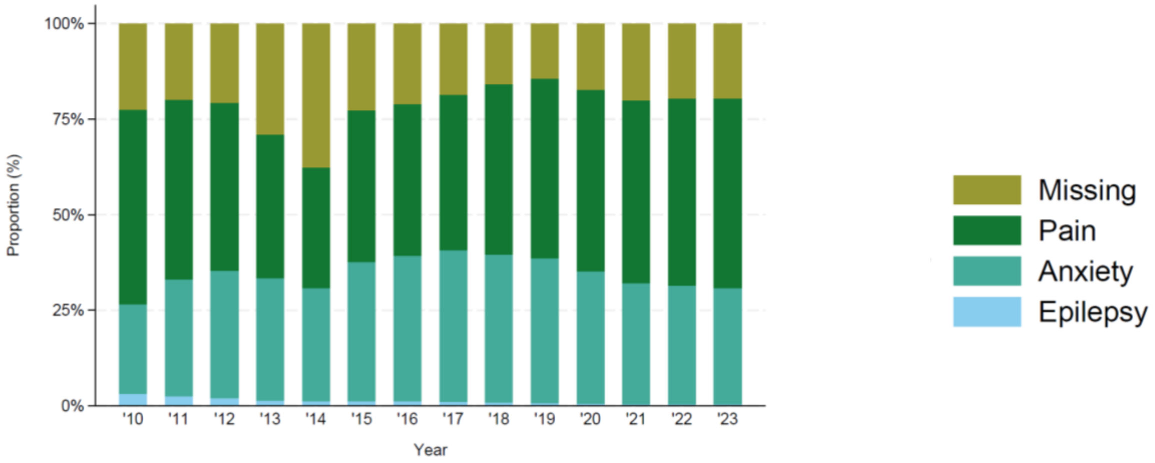

C. Pregabalin

Figure S2

Total amount of gabapentinoids used per year among Danish adults, expressed as the total amount of dispensed defined daily doses (DDDs) at community pharmacies. One DDD correspond to 1,800mg of gabapentin or 300mg of pregabalin.

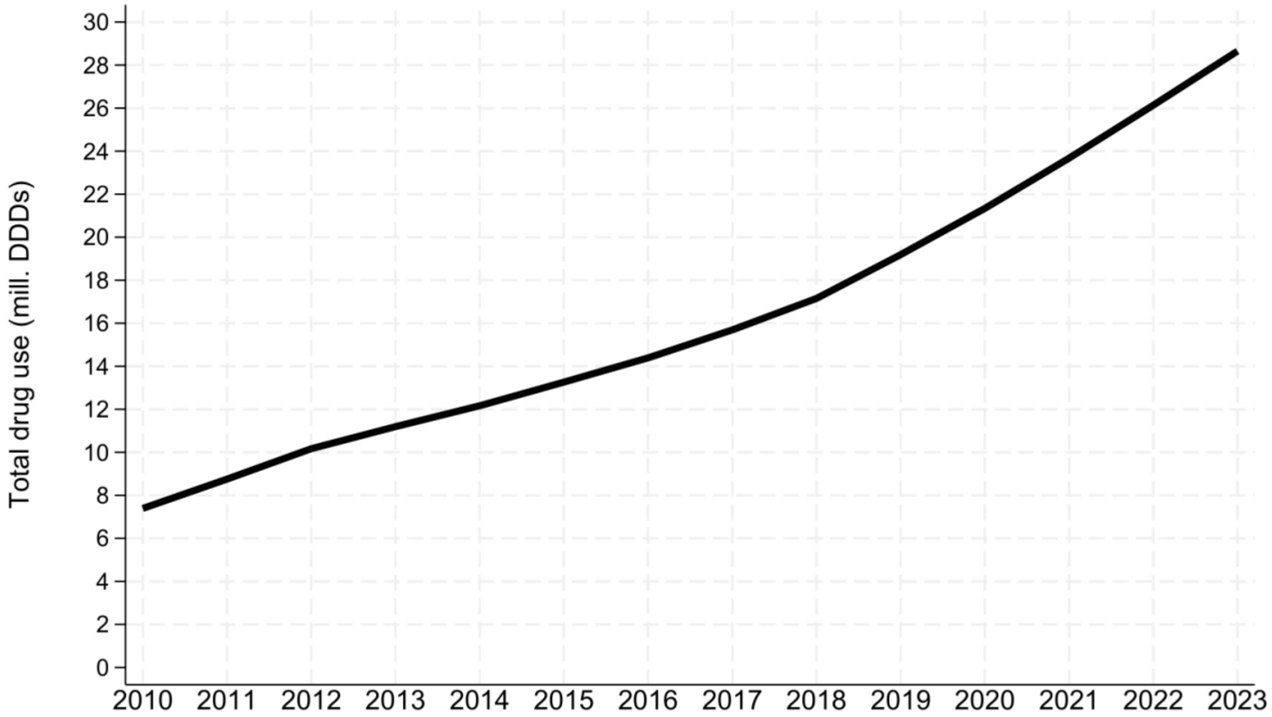

Figure S3

The prevalence proportion per 1,000 persons of all gabapentinoids during 2010 to 2023. Prevalent use was defined as a fill for a gabapentinoids within the corresponding year.

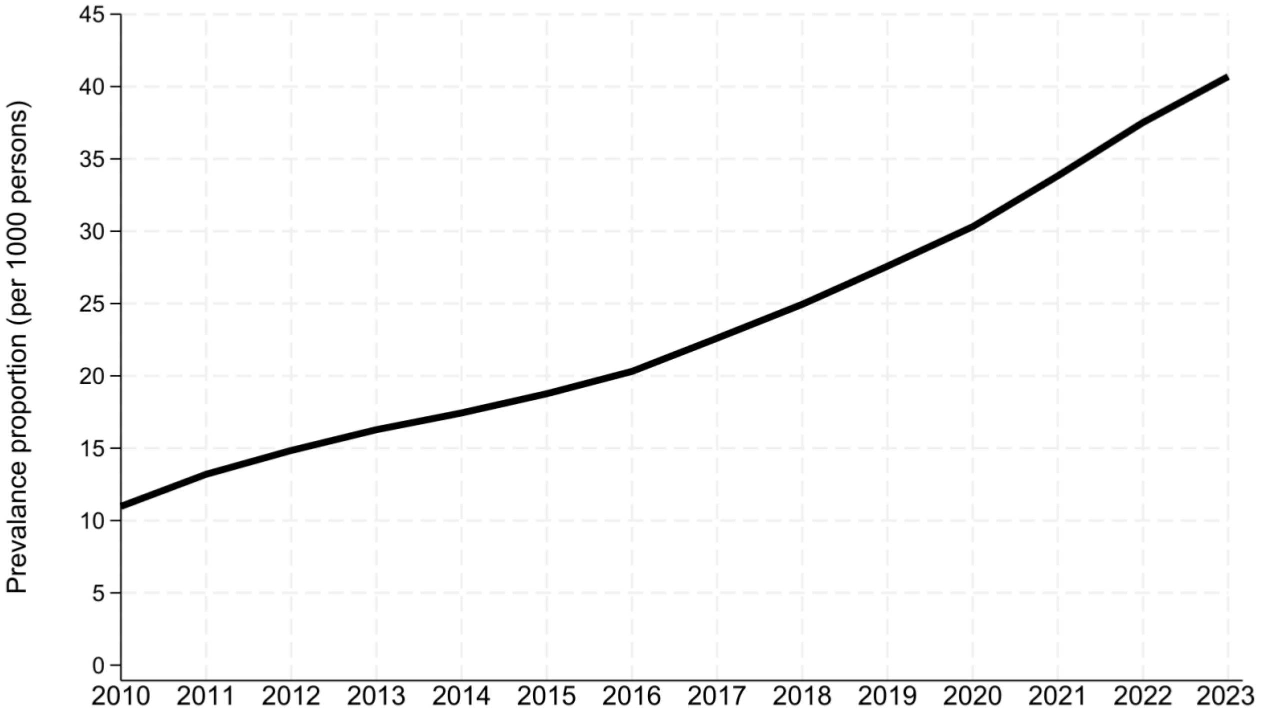

Figure S4

Incidence rates of new users of gabapentinoids during 2010 to 2023. New (incident) use was defined as the first fill for a gabapentinoid in at least five years.

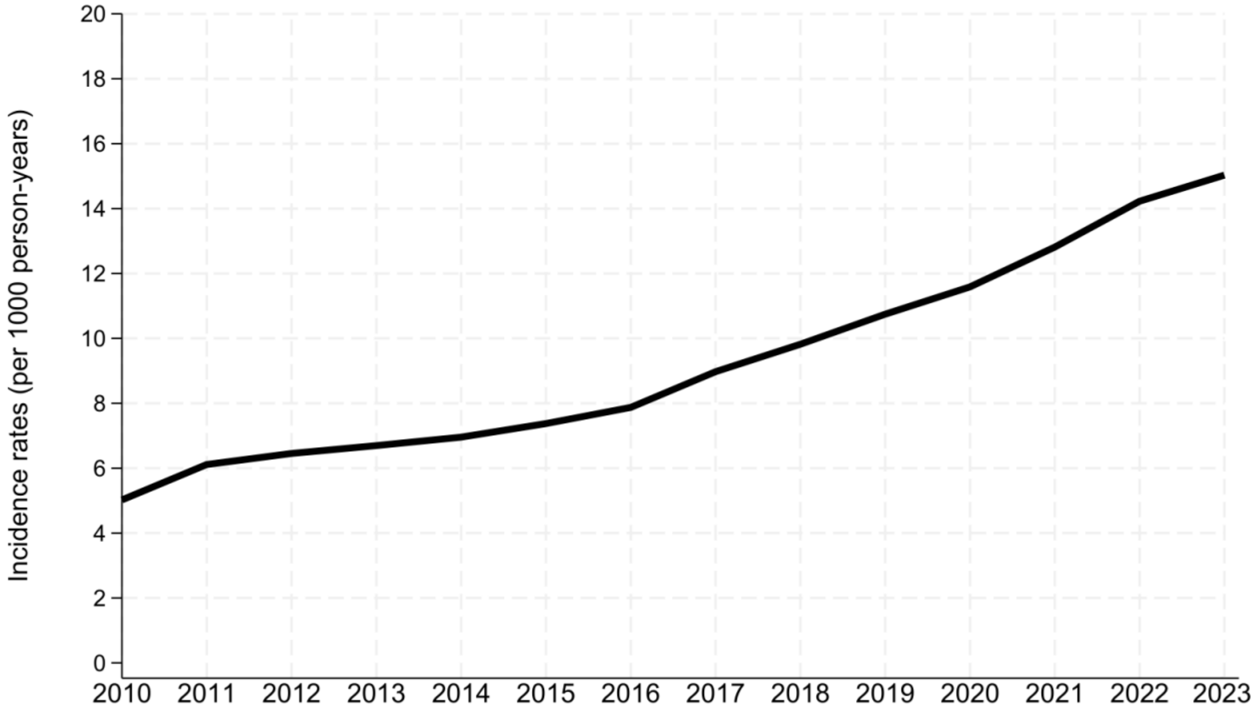

Figure S5

Annual incidence rates of new users of gabapentin during 2010 to 2023. New (incident) use was defined as the first fill of a gabapentinoid in at least five years. New (incident) use of gabapentin with recent use of pregabalin (within five years) was not included as new (incident) gabapentin use.

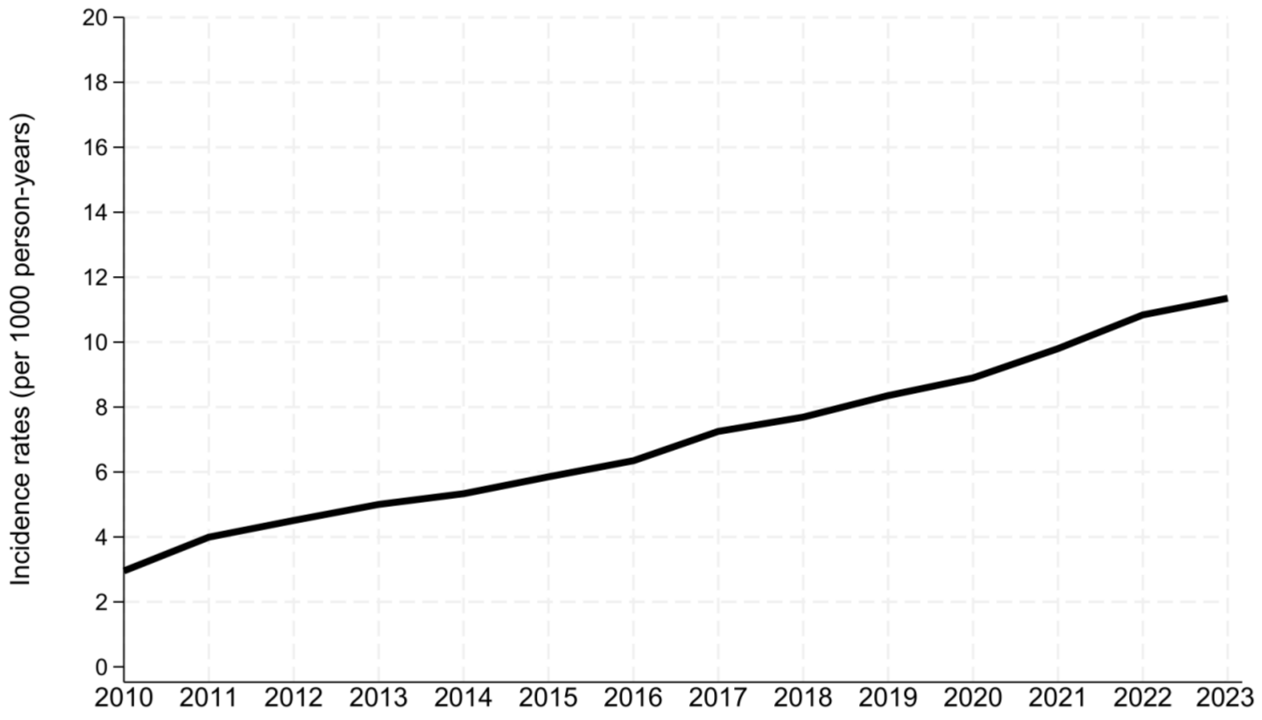

Figure S6

Annual incidence rates of new users of pregabalin during 2010 to 2023. New (incident) use was defined as the first fill of pregabalin in at least five years. New (incident) use of pregabalin with recent use of gabapentin (within five years) was not included as new (incident) pregabalin use.

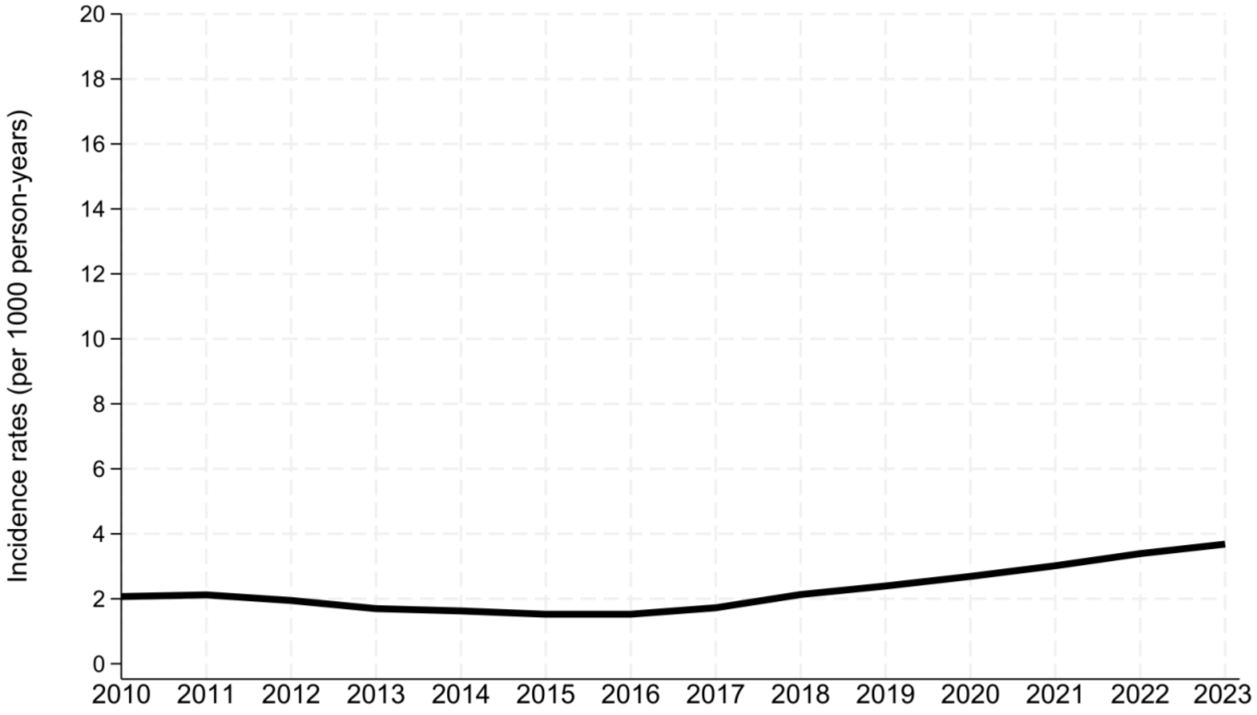

Figure S7

The prevalence proportion per 1,000 persons per year of all gabapentinoids, by sex, during 2010 to 2023. Prevalent use was defined as a fill for a gabapentinoids within the corresponding year.

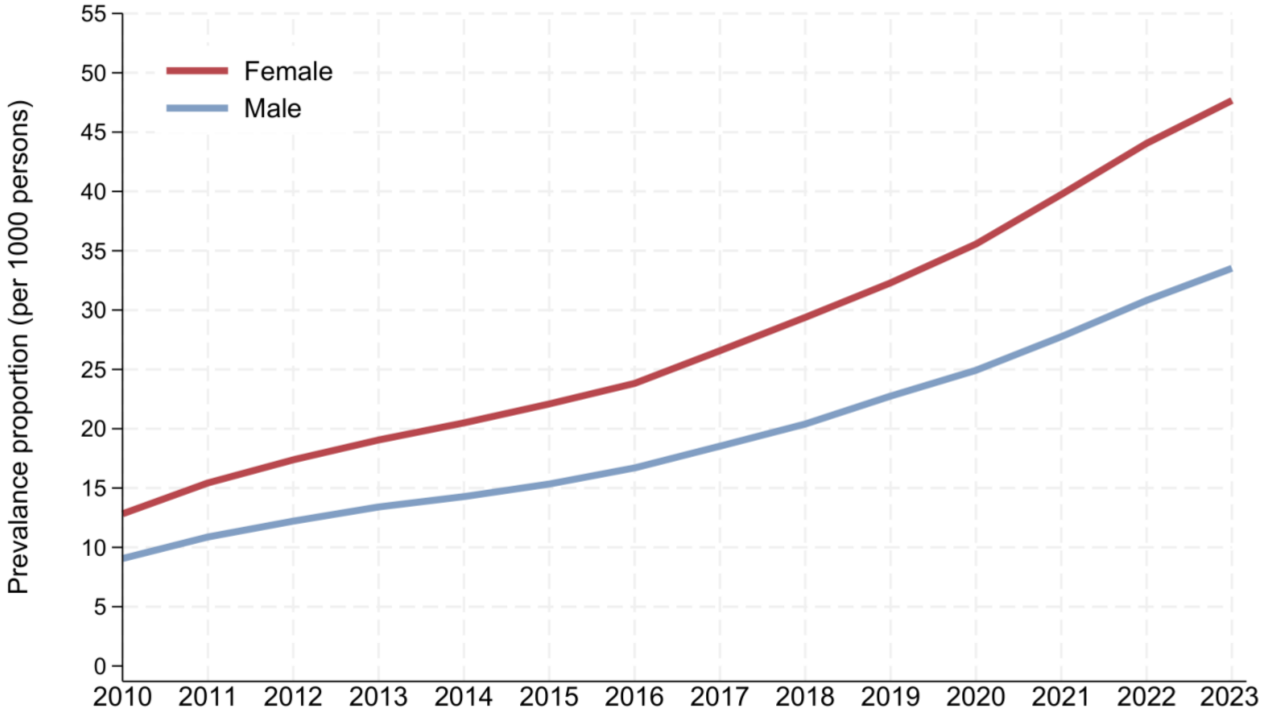

Figure S8

The prevalence proportion per 1,000 persons of gabapentinoids by age and sex during 2010.

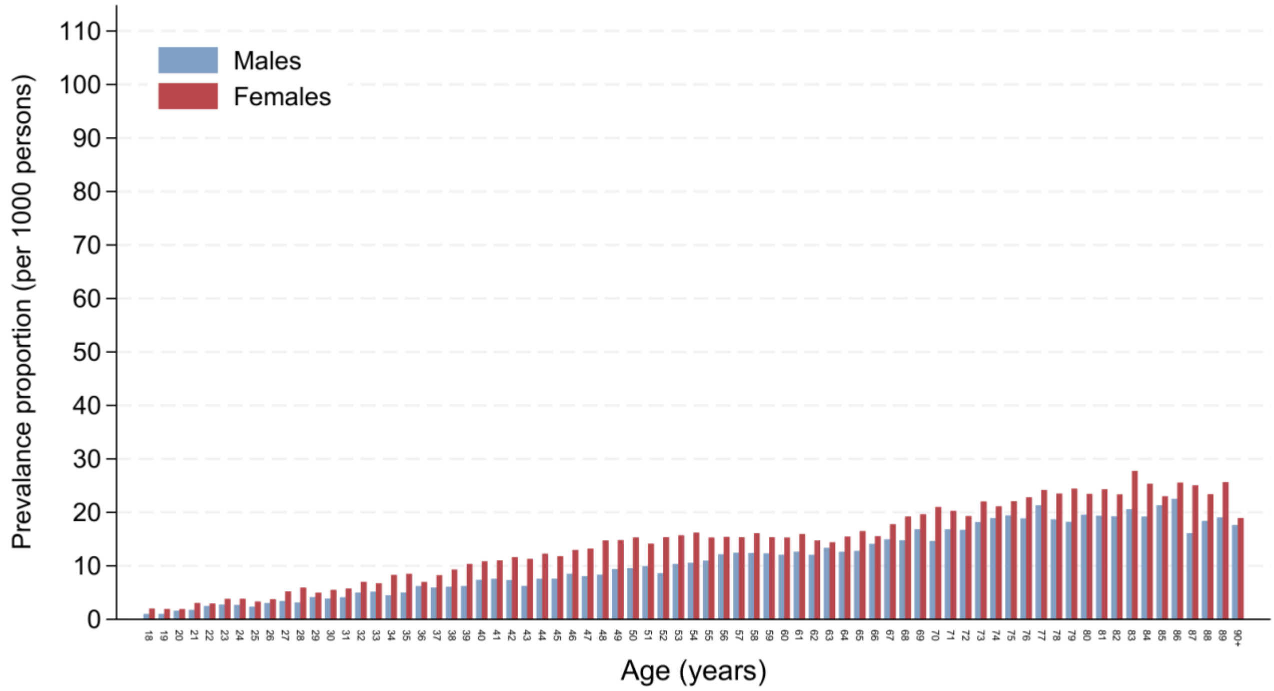

Figure S9

The prevalence proportion per 1,000 persons of gabapentinoids by age and sex during 2017.

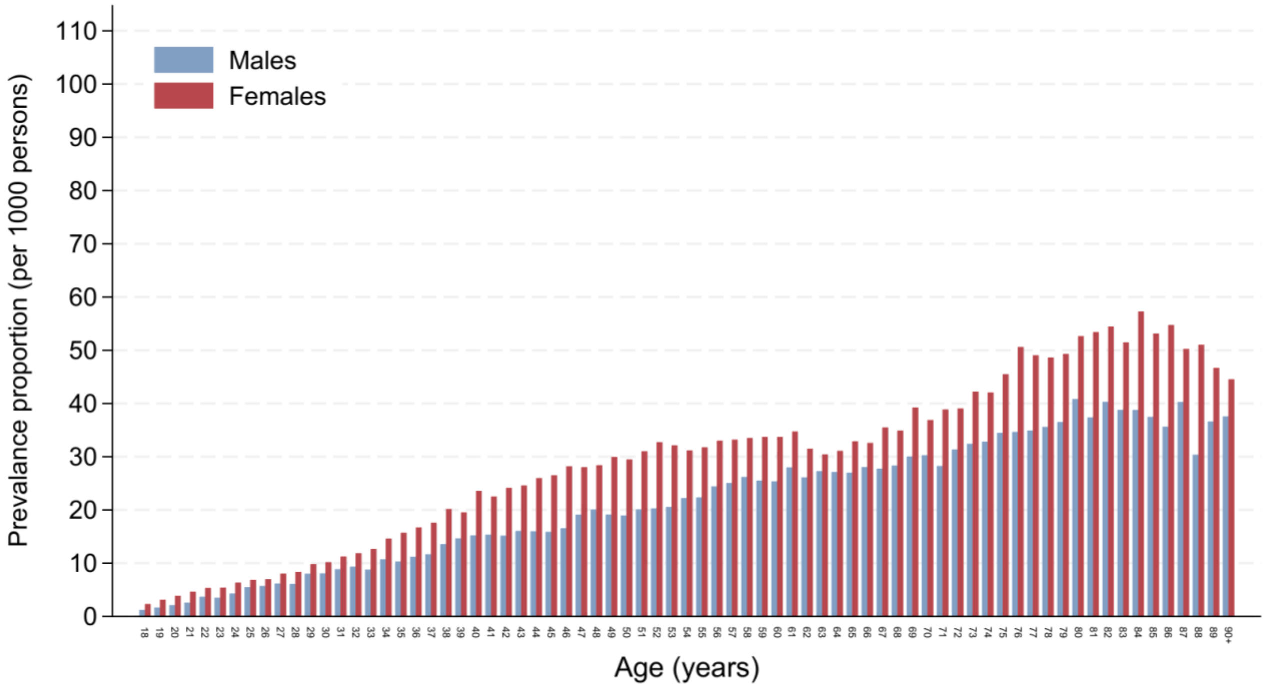

Figure S10

Proportion of gabapentinoids (overall and specified as gabapentin and pregabalin) that were initiated (defined as first prescription in five years for the given patient) and maintained (defined as all other prescriptions) speciality of prescribers annually during 2010 to 2023. Turquoise denotes private practicing specialist prescribers, dark green denotes general practitioners, beige denotes hospital prescribers and yellow denotes unknown prescribers.

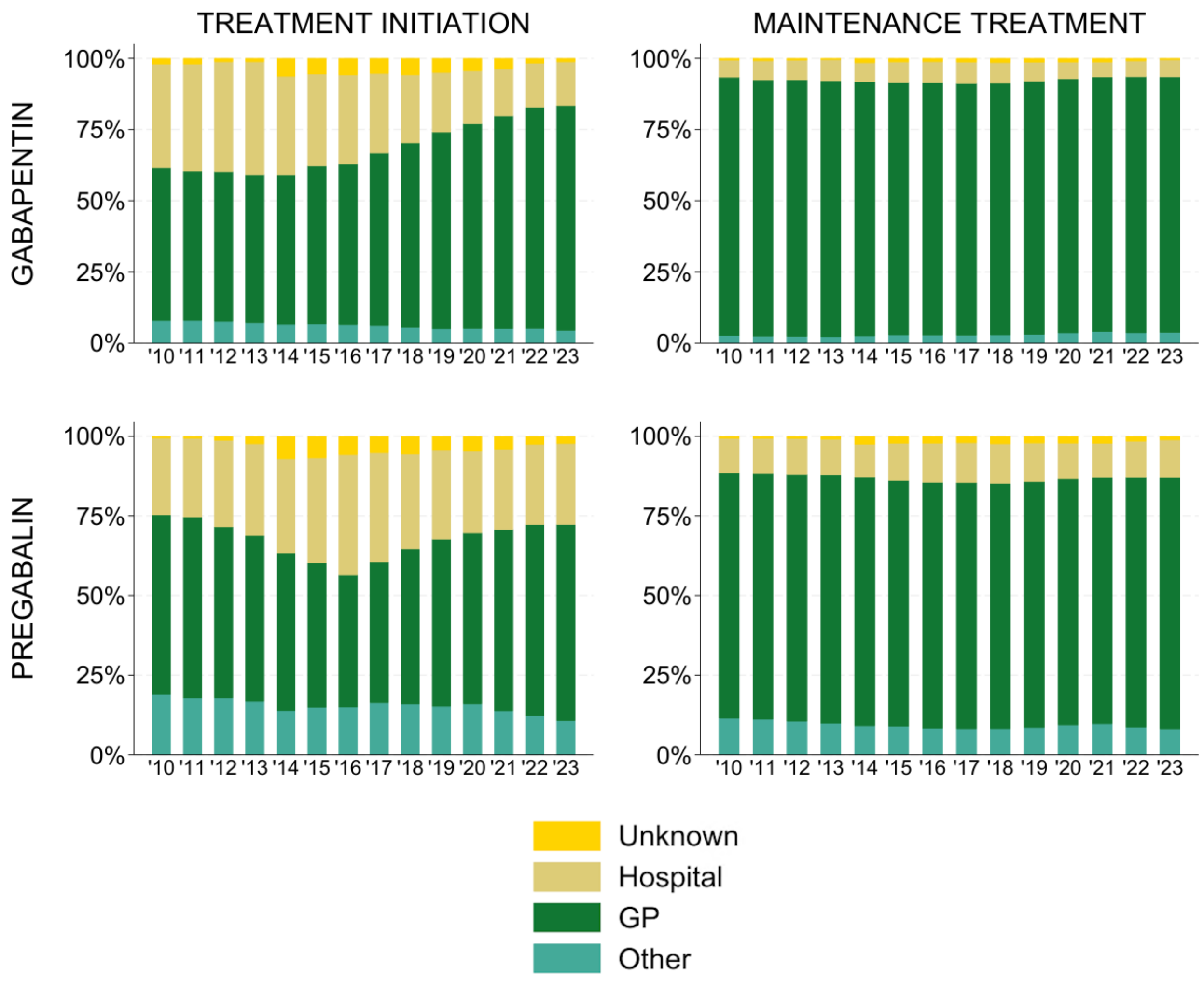

Figure S11

Duration of gabapentinoid therapy specified as the proportion of patients covered (current treatment) since each patient's incident (first in at least five years) prescription during 2010 to 2023, specified by age strata.

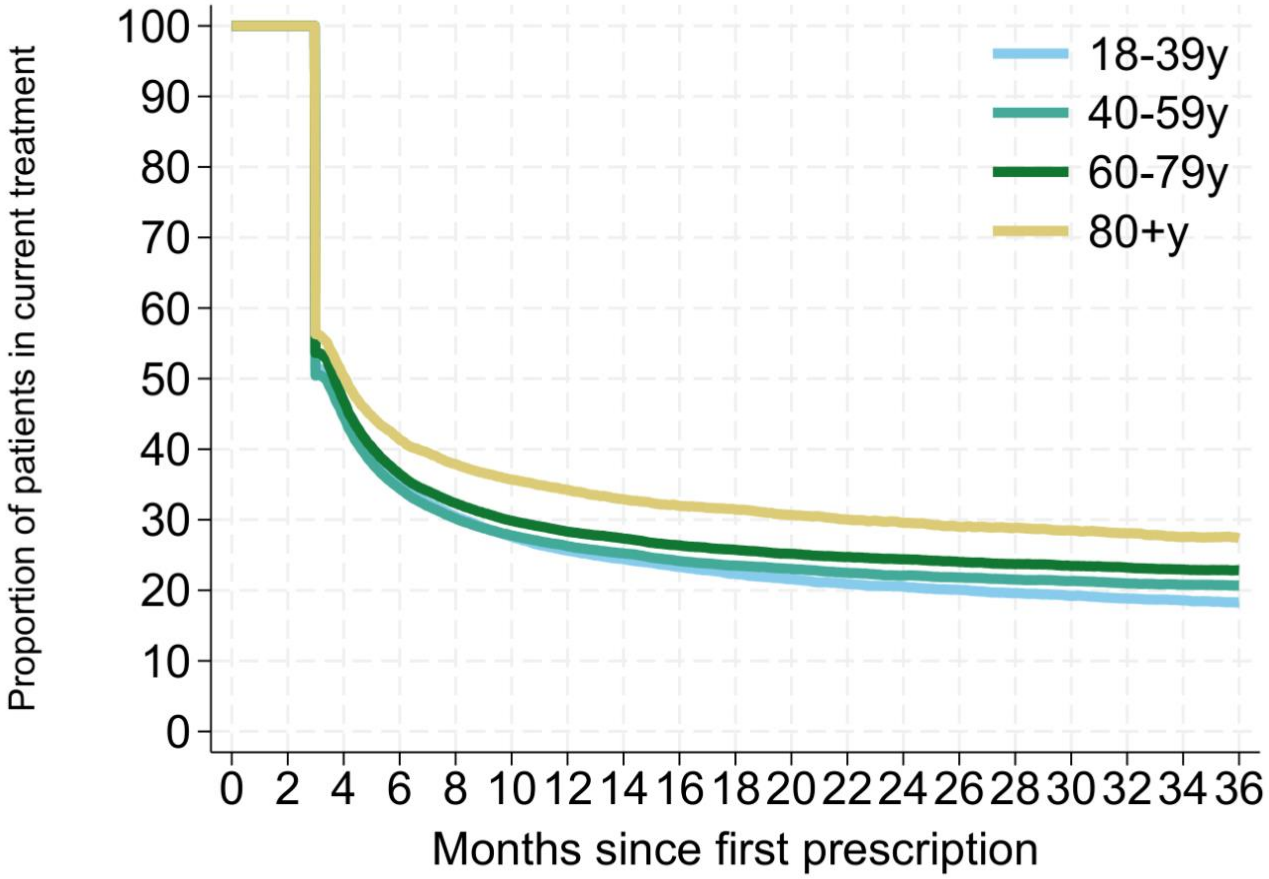

Supplement: Supplementary file 1 — TABLE S1 Codes and definitions. TABLE S2 Characteristics of pregabalin and gabapentin users in 2010, 2017 and 2023, defined as those filling at least 1 gabapentinoid prescription during the respective year. TABLE S3 Translations and grouping of indications for gabapentinoids. FIGURE S1 Proportion of gabapentinoids (overall and specified as gabapentin and pregabalin) by recorded indication 2010–2023. Turquoise denotes anxiety, dark green pain, blue epilepsy and beige missing. FIGURE S2 Total amount of gabapentinoids used per year among Danish adults, expressed as the total amount of dispensed defined daily doses (DDDs) at community pharmacies. One DDD corresponds to 1800 mg of gabapentin or 300 mg of pregabalin. FIGURE S3 The prevalence proportion per 1000 persons of all gabapentinoids during 2010–2023. Prevalent use was defined as a fill for a gabapentinoids within the corresponding year. FIGURE S4 Incidence rates of new users of gabapentinoids during 2010–2023. New (incident) use was defined as the first fill for a gabapentinoid in at least 5 years. FIGURE S5 Annual incidence rates of new users of gabapentin during 2010–2023. New (incident) use was defined as the first fill of a gabapentinoid in at least 5 years. New (incident) use of gabapentin with recent use of pregabalin (within 5 years) was not included as new (incident) gabapentin use. FIGURE S6 Annual incidence rates of new users of pregabalin during 2010–2023. New (incident) use was defined as the first fill of pregabalin in at least 5 years. New (incident) use of pregabalin with recent use of gabapentin (within 5 years) was not included as new (incident) pregabalin use. FIGURE S7 The prevalence proportion per 1000 persons per year of all gabapentinoids, by sex, during 2010–2023. Prevalent use was defined as a fill for a gabapentinoids within the corresponding year. FIGURE S8 The prevalence proportion per 1000 persons of gabapentinoids by age and sex during 2010. FIGURE S9 The prevalence proportion per 1000 [file BCP-91-2515-s001.pdf]
